# High place phenomenon: prevalence and clinical correlates in two German samples

**DOI:** 10.1186/s12888-020-02875-8

**Published:** 2020-09-30

**Authors:** Tobias Teismann, Julia Brailovskaia, Svenja Schaumburg, André Wannemüller

**Affiliations:** Mental Health Research and Treatment Center, Ruhr-Universität Bochum, Massenbergstraße 9-13, 44787 Bochum, Germany

**Keywords:** High place phenomenon, Suicide ideation, Depression, Anxiety, Anxiety sensitivity

## Abstract

**Background:**

The high place phenomenon, that is, a sudden urge to jump when in a high place, is an experience known to many people, that has rarely been studied. The present study aimed to assess the prevalence of the high place phenomenon in a non-clinical and a clinical German sample. Furthermore, clinical correlates of the experience were assessed.

**Methods:**

The study sample comprised 276 participants (67% female; *M*_age_ = 32.08, *SD*_age_ = 10.73) who took part in an online assessment and 94 patients (73.4% female; *M*_age_ = 49.26, *SD*_age_ = 13.32) suffering from clinically relevant fear of flying. Participants filled out questionnaires on experiences with the high place phenomenon, depression, anxiety, suicide ideation and anxiety sensitivity.

**Results:**

The high place phenomenon was known to nearly 60% of the online sample and to 45% of the patient sample. Suicide ideation as well as anxiety sensitivity were positively associated with experiences with the high place phenomenon in the online sample. Depression, anxiety and suicide ideation were unrelated to experiences with the phenomenon in the patient sample.

**Conclusion:**

The high place phenomenon is commonly reported by (lifetime/current) suicide ideators. However, it is also a common experience in individuals who have never suffered from suicide ideation. It is therefore cautioned not to interpret such experiences as an expression of a hidden death wish.

## Background

Many people are familiar with the experience of a sudden urge to jump when in a high place, that is, when standing on a bridge or a viewing platform. On the Internet this experience is described and discussed under the term *call of the void*, while Hames and colleagues [1] have coined the term *high place phenomenon*. Although it is an experience known to many people, the phenomenon has rarely been studied.

In the only study published on the phenomenon by now, Hames et al. [1] investigated a sample of 432 undergraduate college students. They could show that over 50% of participants who have never suffered from suicide ideation in their lifetime, reported to have experienced the phenomenon at least once in their lives. Furthermore, over three-quarters of lifetime suicide ideators reported experiencing the urge to jump from a window of a tall building or off a bridge or building. Though experiences with the high place phenomenon were strongly associated with current and lifetime suicidal ideation as well as severity of current depressive symptoms, the finding that 50% of lifetime non-ideators were familiar with the sudden urge to jump from a high place indicates that the experience in itself is not a sign of psychopathology and does not necessarily express a death wish. The phenomenon might be comparable to intrusive thoughts, that is, unpleasant, fleeting and ego-dystonic thoughts or images about for example sexual, violent and/or blasphemous content which are universal among people [2], but can also be a feature of an obsessive-compulsive disorder.

Hames et al. [1] propose that – in non-suicidal individuals – the experience of the high place phenomenon stems from the misinterpretation of a safety or survival signal: “Put simply, when an individual stands on a high place, his or her fear circuitry might react to the potential danger in the situation by sending a rapid signal such as, “Back up, you might fall.” This “safety signal” is intended to keep the person alive and out of danger, and it is fired so quickly that the person backs away from the edge, often without being fully aware of why he or she did this. It is not until moments later, when the person tries to understand his or her behavior, that the individual’s slower perceptual system kicks in and potentially misattributes the safety signal (“Getting too close, back up”) to a death wish involving heights” ([1], p. 1119).

In line with the hypothesis that a misinterpreted safety signal is at the core of the high place phenomenon, Hames et al. [1] suggest that individuals who tend to be more sensitive to such safety signals are more likely to report experiencing the phenomenon. Indeed, the authors could show that anxiety sensitivity, that is, the tendency to be fearful of anxiety-related symptoms and arousal sensations [3], was associated with experiences with the high place phenomenon – especially in students exhibiting low levels of concurrent suicide ideation. Yet, no study so far has studied the prevalence and correlates of experiences with the high place phenomenon in patients suffering from an anxiety disorder, that is, individuals who are especially prone to react sensitively to safety signals [4].

On this background, the current study had two aims: The first aim was to replicate the findings by Hames et al. [1] regarding prevalence and clinical correlates of the high place phenomenon in a more diverse sample of German adults recruited online. The second aim was to assess experiences with the high place phenomenon in a sample of anxiety patients suffering from flight phobia. In line with previous findings, we expected the high place phenomenon to be known to at least 50% of all participants – irrespective of lifetime or current suicide ideation. Finally, we expected a positive association between anxiety sensitivity and knowledge of the high-place phenomenon.

## Methods

### Participants and procedure

Data was derived from two samples in Germany.

#### Sample 1 (online sample)

The first sample comprised *N* = 276 participants (67% female; *M*_age_ = 32.08, *SD*_age_ = 10.73, range: 18–64 years) who took part in an online assessment between February and June 2020. One-hundred twenty-one participants (43.8%) reported lifetime suicide ideation, 69 participants (25%) reported some suicide ideation in the last 4 weeks (SSEV- score ≥ 1) and 26 participants (9.4%) indicated that they had attempted suicide at least once in their lifetime (range: 1–6). All participants were of Caucasian decent.

Data was collected through an anonymous online survey using the SoSci-server (https://www.soscisurvey.de/). Participants were recruited through postings at local universities. In order to take part in the study, participants had to be at least 18 years old and to give their consent to participation at the beginning of the study.

#### Sample 2 (patient sample)

The second sample comprised *N* = 94 participants (73.4% female; *M*_age_ = 49.26, *SD*_age_ = 13.32, range: 22–79 years) who completed the High Place Phenomenon Index [1] at a follow-up assessment after a one-day intensive CBT-treatment for flight phobia (cf. [5]) between August and October 2019. All participants suffered from clinically relevant fear of flying before treatment: 90.3% suffered from flight phobia, 8.6% from agoraphobia and 1.1% from height phobia according to the Diagnostic and Statistical Manual for Mental Disorders (DSM-5 [6]). At the follow-up assessment 80 patients (85.1%) were available for a second diagnostic interview. Of those *n* = 44 (55%) were classified as completely remitted. Diagnoses were assigned in individual diagnostic sessions conducted either by licensed psychotherapists or post-graduate clinical psychologists using a structured clinical interview (Short Interview for Mental Disorders, Mini-DIPS-OA [7]). Very good inter-rater reliability for anxiety disorders (κ = .94) has been reported for the long version of the DIPS [8]. Two participants (2.1%) indicated some suicide ideation (BDI-suicide item ≥1) in the week before the assessment. All participants were of Caucasian decent.

Treatment and assessment took place at a university outpatient clinic in the Ruhr Area in Germany. In order to take part in the study, participants had to be at least 18 years old, suffer from a clinically relevant fear of flying, that is, suffer from a phobic disorder, and to give their consent to participation at the beginning of the study.

Prior to assessments, participants in both studies were informed about the purpose of the study, the voluntary nature of their participation, data storage and security. Both studies were approved by the responsible Ethics Committee.

### Measures

The investigations of the two samples were planned independently. Therefore, not all questionnaires were used in both sub-samples. In the following, it is indicated which questionnaire was used in which sample.

#### High place phenomenon index (HPPI [1];)

The HPPI assesses with three items how frequently participants have experienced the high place phenomenon in their lifetime, using a 6-point Likert type scale ranging from (0) never to (5) always: Item 1: *When standing on the edge of a tall building or walking on a bridge, have you ever had the urge to jump?* Item 2: *When you see a tall building or are walking on a bridge, have you ever thought about what it would be like to jump off of it?* Item 3: *When you are inside a tall building have you ever imagined jumping out a window?* The original scale evidenced good internal consistency: *α* = .85. The German version of the HPPI was developed by means of a translation-back-translation procedure according to relevant guidelines for the translation of psychometric instruments [9]. Internal consistencies of the German HPPI were good in the online sample (*α* = .84) as well as in the patient sample (*α* = .86).

#### Depression anxiety stress scales (DASS [10];)

The DASS is a 21-item questionnaire measuring depressive mood, anxiety and chronic tension/stress during the past week (“I was aware of dryness of my mouth”; “I couldn’t seem to experience any positive feeling at all.”) All items are rated on a 4-point (0–3) scale. Internal consistency (coefficient alpha) for the DASS has been shown to be good (range of *α* = 0.88–0.96 [11];). In the current study, only the DASS-Depression and the DASS-Anxiety subscales were used. Internal consistencies of both subscales were good: *α* = .91 (DASS-Depression) and *α* = .84 (DASS-Anxiety) in the online sample, and sufficient in the patient sample: *α* = .77 (DASS-Depression) and *α* = .71 (DASS-Anxiety).

#### Anxiety sensitivity index (ASI [12];)

The ASI is a 16-item self-report inventory designed to measure the degree to which individuals are concerned about the potential negative effects of experiencing anxiety symptoms (“It scares me when I feel faint”, “Unusual body sensations scare me”). Respondents are asked to indicate the degree to which each item applies to them using a 5-point Likert type scale ranging from (0) very little to (4) very much. The scale has been found to have strong internal consistency and test-retest reliability (e.g., [13]). The ASI was only used in the online sample. Internal consistency was good within this sample: *α* = .88.

#### Fear of flying scale (FFS [14, 15];)

Fear of Flying (FoF) was assessed using the German version of the FFS consisting of 21 items with 5-point Likert scales (0 = no fear; 4 = very strong fear). The instrument provides a dimensional assessment of FoF severity and has shown to have good internal consistency: *α* = .90 [15]. The FFS was only used in the patient sample. Internal consistency of the FFS was excellent within this sample: *α* = .96.

#### Suicide ideation and behavior scale – suicide ideation (SSEV-SI [16];

The SSEV-SI assesses with eight items the frequency of suicide ideation in the past 4 weeks (e.g., “During the past four weeks, … I thought it would be better if I wasn’t alive, … I’ve been thinking about killing myself, … I have seriously considered killing myself”). All items are answered on a 6-point Likert scale ranging from “0 = *never*” to “4=*many times every day*”, with higher scores indicating greater severity of suicidal ideation. Internal consistency was good in the current online sample (*α* = .87). Occurrence (“In the course of my life I have tried to kill myself (and I really wanted to die)”) and number of lifetime suicide attempts (“How many times have you tried to kill yourself?”) are assessed with two further SSEV-items. Finally, lifetime suicide ideation was assessed using a screening item from the Perseverative Thoughts about Suicide Questionnaire [17]: “In my lifetime I have seriously considered suicide”. The SSEV was only used in the online sample.

#### Beck depression inventory – suicide item (BDI-SI [18];)

Suicide ideation was measured using the respective item from the BDI (Item 9) in the patient sample. The suicide item of the BDI has repeatedly been used in clinical studies [19] and has been shown to significantly correlate with the Beck Scale for Suicide Ideation [20].

### Statistical analyses

Statistical analyses were conducted with SPSS 24. Descriptive statistics and zero-order bivariate correlations between the investigated variables were calculated. Furthermore, group differences between (lifetime/current) suicide ideators and non-ideators were investigated using t-tests. To identify significant predictors of experiences with the high place phenomenon, hierarchical regression analyses – including age, gender, lifetime suicide ideation, depressive symptoms (DASS-D), anxiety symptoms (DASS-A) and anxiety sensitivity (ASI) as independent variables and HPPI scores as dependent variable – were calculated using the online sample. There was no violation of the multicollinearity assumption as all values of tolerance were > .25, and all variance inflation factor values were < 5.

## Results

### Descriptive statistics, correlations and group differences

The high place phenomenon was known to *n* = 165 (59.8%) of the online sample (HPPI-score ≥ 1). More precisely, the high place phenomenon was known to *n* = 70 (45.2%) of non-ideators, to *n* = 95 (78.5%) of lifetime suicide ideators and to *n* = 56 (81.2%) of current suicide ideators within the online sample. Lifetime non-ideators (*M* = 1.00, *SD* = 1.49) and lifetime suicide ideators (*M* = 2.95, *SD* = 2.47) differed in HPPI-scores, *t*(274) = − 8.11, *p* < .001; same as current non-ideators (*M* = 1.37, *SD* = 1.87) and current suicide ideators (*M* = 3.30, *SD* = 2.48) differed in HPPI-scores, *t*(274) = − 6.80, *p* < .001. Finally, lifetime suicide attempters (*M* = 4.21, *SD* = 2.64) exhibited higher HPPI-scores than lifetime suicide ideators (without a history of suicide attempts, *M* = 2.65, *SD* = 2.34), *t*(119) = − 2.80, *p* < .01.

Of the patient sample, 43 participants (45.7%) indicated to have experience with the high place phenomenon. Since only two patients reported current suicide ideation, no group comparisons were conducted within this sample. Descriptive statistics for each measure and correlations are presented in Table 1. Correlation analyses indicated that all study variables correlated positively with each other, that is, experiences with the high place phenomenon were associated with depression, anxiety and anxiety sensitivity in the online sample. However, there were no significant associations between study variables in the patient sample.

**Table 1:** Means, standard deviations and correlations of study variables

	HPPI (Online Sample)				HPPI (Patient sample)	
		Total	non-ideators (*n* = 155)	lifetime ideators (*n* = 121)		Total (*n* = 94)
	*M* (*SD*)	*r*	*r*	*r*	*M* (*SD*)	*r*
**DASS-D**	5.10 (5.81)	.412**	.190*	.290**	1.48 (2.18)	.047
**DASS-A**	3.47 (3.47)	.374**	.228**	.240**	1.77 (2.35)	.019
**ASI**	32.03 (9.77)	.417**	.287**	.343**	–	–
**SSEV-SI**	1.14 (2.84)	.393**	.175*	.306**	–	–
**BDI-SI**	–	–	–	–	0.2 (0.14)	.185
**FFS**	–	–	–	–	27.6 (16.85)	.064
**HPPI**	1.85 (2.20)	–	–	–	1.29 (2.16)	–

*Note*. *M* Mean, *SD* Standard Deviation; *r* Pearson correlation, *ASI* Anxiety Sensitivity Index, *BDI-SI* Beck Depression Inventory-Suicide Item, *DASS-A* Depression Anxiety Stress Scales – Anxiety Subscale, *DASS-D* Depression Anxiety Stress Scales – Depression Subscale, *FFS* Fear of Flying Scale, *HPPI* High Place Phenomenon Index, *SSEV-SI* Suicide Ideation and Behavior Scale – Suicide Ideation

**p < .01, *p < .05

### Prediction of experiences with the high place phenomenon

The results of the hierarchical regression analyses – using data from the online sample – are shown in Table 2. Experiences with the high place phenomenon were predicted by lifetime suicide ideation and anxiety sensitivity. None of the other variables predicted experiences with the high place phenomenon within the online sample.

**Table 2:** Multiple linear models for the prediction of experiences with the high place phenomenon (online sample)

	Model 1			Model 2			Model 3		
	B	T	p	B	T	p	B	T	p
Age	0.01	−1.16	.247	0.01	−0.96	.336	0.00	−0.72	.472
Gender	0.47	1.70	.090	0.35	1.37	.170	0.45	1.87	.062
Ideator vs. Non-Ideator	–	–	–	**1.92**	**7.96**	**.000**	**1.21**	**4.65**	**.000**
DASS-D	–	–	–	–	–	–	0.06	1.75	.081
DASS-A	–	–	–	–	–	–	0.03	0.70	.480
ASI	–	–	–	–	–	–	**0.05**	**3.42**	**.001**
**Model**	Adj. R^2^ = .008 *F*(2,273) = 2.12 *p* = .122			Adj. R^2^ = .193 *F*(3,272) = 22.90 *p* ≤ .000			Adj. R^2^ = .284 *F*(6, 269) = 19.14 *p* ≤ .000		

*Note*. *ASI* Anxiety Sensitivity Index, *DASS-A* Depression Anxiety Stress Scales – Anxiety Subscale, *DASS-D* Depression Anxiety Stress Scales – Depression Subscale

## Discussion

In the present study, experiences with the high place phenomenon were examined in two German samples. The phenomenon is well known to persons who suffer or have suffered from suicide ideation: About 80% of (lifetime/current) suicide ideators are familiar with the experience of a sudden urge to jump when in a high place. In the context of a suicidal crisis, the high place phenomenon represents only one of many different experiences; as reports of a sudden urge to steer the car into oncoming traffic, or to jump in front of an approaching train, or a sudden urge to injure oneself with a knife, are no rarity in clinical practice as well as in internet chats.

However, the phenomenon is not only well-known to individuals suffering from suicide ideation, but also to individuals not suffering from (lifetime/current) suicide ideation. In the current study, about 45% of participants who had not suffered from suicide ideation in their lifetime, reported experiencing the phenomenon at least once in their lives. Almost identical prevalence figures were reported by Hames and colleagues [1], who investigated the phenomenon in an American student sample. Taken together, the findings clearly indicate that the high place phenomenon is a common phenomenon that is unlikely to refer to a hidden death wish. In fact, it was shown that experiences with the high place phenomenon are associated with increased anxiety sensitivity: In a hierarchical regression analysis, anxiety sensitivity – as well as lifetime suicide ideation – proved to be the sole predictors for experiences with the high place phenomenon. The tendency to be fearful of anxiety-related symptoms and arousal sensations thus seems to be particularly associated with the high place phenomenon. This finding supports the assumption of Hames and colleagues [1] that the high place phenomenon stems from a misinterpretation of a safety or survival signal (“Back up, you might fall”) and therefore is more likely to be reported by individuals who tend to be more sensitive to such safety signals (i.e., individuals exhibiting heightened levels of anxiety sensitivity). However, neither the present findings nor the findings of Hames et al. [1] can be interpreted as stringent proof of this assumption. The cross-sectional design of both studies does not allow fine-grained analysis of temporal patterns. It may therefore be the case that individuals exhibiting higher levels of anxiety sensitivity simply remember experiences with the high place phenomenon better in retrospect, as they experienced them as more threatening at the time. Experimental studies, where anxiety sensitivity is recorded in advance and participants are then confronted with height situations, could provide more precise information. Additional physiological measurements would enable the inclusion of physical correlates of the high place phenomenon.

Eventually, the question arises to what extent the coincidence of suicide ideation and the high place phenomenon represents a risk for suicidal behavior: Is a transition from suicide ideation to suicidal behavior more likely if an individual experiences sudden urges to jump from high places? On the one hand, experiences with the high place phenomenon were more common to lifetime suicide attempters than to lifetime suicide ideators in the current study. On the other hand, however, this finding does not imply that suicide attempts immediately follow sudden urges to harm oneself. In general, the idea that people die by suicide “on a whim” has been disputed [21]: It is true that the time span between a decision to die by suicide and its implementation is often short (e.g., [22]), nonetheless, suicide has regularly been considered far in advance (e.g., [23]). Future studies should strive to clarify the role of sudden urges to jump in the decision process regarding suicide. Already now, questions on suicidal impulses should be included in suicide risk assessments.

Of note, findings described up to this point were only evident in the online sample and not in the patient sample. In fact, about 45% of the participants in the patient sample also stated that they have experience with the high place phenomenon. Yet, no significant associations between the high place phenomenon and anxiety or suicide ideation were found in the patient sample. This may be related to the fact that the vast majority of participants in the patient sample suffered from a specific flight phobia which is (a.) very specific and isolated (b.) causes comparably low symptom distress (i.e., low variance) and (c.) is to a lesser extent characterized by anxiety sensitivity: At the core of flight phobia is the fear that the plane might crash and not the threat of certain bodily symptoms [24]. Only less than 10 % of the participants were diagnosed with agoraphobia, a disorder that has been evidenced to be associated with enhanced anxiety sensitivity [25]. It would therefore be advisable to give priority to individuals suffering from panic disorders with/without agoraphobia and/or height phobia in future studies on the high place phenomenon (cf., [4, 26]).

The results of the current study should be interpreted with consideration of the following limitations. First, lifetime suicide ideation and lifetime history of suicide attempts were not assessed in the patient sample. Therefore, it was impossible to replicate the findings from the online sample within the patient sample. Furthermore, suicide ideation was only assessed with the respective item of the BDI instead of a more comprehensive method to assess suicide ideation in the patient sample. This might have led to an underestimation of suicidality within the patient sample. However, it has to be noted that symptom distress was generally low within this sample. Second, the HPPI was part of the follow-up assessment in the patient sample. At the post-treatment follow-up assessment, roughly half of the patients no longer met criteria for an anxiety disorder. Although the HPPI asks for lifetime experience with the high place phenomenon, the possibility that successfully treated participants are less likely to recall anxiety provoking experiences cannot be ruled out completely. Third, the online sample was collected during the COVID-19 pandemic. It might therefore be that higher stress, anxiety and depression levels were experienced by participants (cf., [27]) - possibly leading to a better recall of anxiety provoking experiences. However, since Hames et al. [1] found comparable prevalence rates of the high-place phenomenon, a recall bias related to stress is unlikely. Fourth, the use of a cross-sectional research design and a sample comprised of Caucasians only limits the generalizability of the results and the discussion of temporal/causal relationships between study variables. In general, a representative population sample is be necessary to make a definite statement on the prevalence of the high place phenomenon.

## Conclusions

The current results point out that the high place phenomenon is a widespread experience that can be associated with suicide ideation/behavior but is not necessarily a sign of a hidden death wish.

## Data Availability

All relevant data are reported within the paper. Analyzed data are available from the corresponding author on reasonable request.

## Abbreviations

ASI: Anxiety Sensitivity Index
BDI: Beck Depression Inventory
DASS: Depression Anxiety Stress Scales
FFS: Fear of Flying Scale
FoF: Fear of Flying
HPPI: High Place Phenomenon Index
SSEV-SI: Suicide Ideation and Behavior Scale – Suicide Ideation
